# Human Infection from Avian-like Influenza A (H1N1) Viruses in Pigs, China

**DOI:** 10.3201/eid1807.120009

**Published:** 2012-07

**Authors:** Huanliang Yang, Chuanling Qiao, Xu Tang, Yan Chen, Xiaoguang Xin, Hualan Chen

**Affiliations:** Author affiliation: State Key Laboratory of Veterinary Biotechnology–Harbin Veterinary Research Institute, Harbin, People’s Republic of China

**Keywords:** swine influenza virus, pigs, avian-like H1N1, human infection, viruses, People’s Republic of China

## Abstract

In investigating influenza in an immunodeficient child in China, in December 2010, we found that the influenza virus showed high sequence identity to that of swine. Serologic evidence indicated that viral persistence in pigs was the source of infection. Continued surveillance of pigs and systemic analysis of swine influenza isolates are needed.

Humans have been infected with avian-like swine influenza A (H1N1) viruses (SIVs) several times since the first case was diagnosed in Switzerland in 1986 (1). These cases generally occur in persons who have direct exposure to pigs (2–4). On December 31, 2010, a 3-year-old boy in rural Jiangsu Province, People’s Republic of China, who had chronic renal disease (for which he was given long-term steroid treatment), sought care with influenza-like symptoms. Laboratory tests at the Chinese Center for Disease Control and Prevention yielded a positive result for European avian-like A (H1N1) SIV, indicating that the European avian-like SIV also caused human infection in the Asia-Pacific region.

## The Study

**Figure 1:**
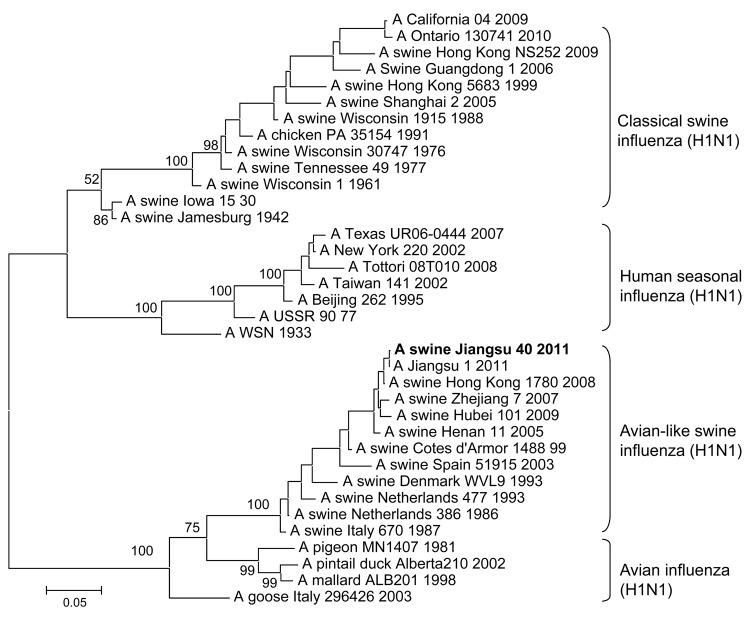
Phylogenetic tree of selected swine, human, and avian H1 hemagglutinin 1 sequences. An unrooted phylogenetic tree was generated by the distance-based maximum-likelihood method by using MEGA5 software (www.megasoftware.net). Bootstrap values were calculated on the basis of 1,000 replications; A/swine/Jiangsu/40/2011 is in **boldface**. Scale bar indicates nucleotide substitutions per site.

After notification of the boy’s infection from the Ministry of Health, we performed active public health surveillance to locate the origin of the infection. A total of 60 nasal swab specimens were collected from pigs at the patient’s family farm and a local slaughterhouse. Each swab was placed in 2 mL of minimal essential medium supplemented with penicillin (2,000 U/mL) and streptomycin (2,000 U/mL). Virus was isolated by using 10-day-old specific pathogen-free embryonated chicken eggs. Hemagglutinin (HA) and neuraminidase (NA) subtypes were determined as described (5). Three A (H1N1) SIVs were obtained, including 2 isolates from pigs in the slaughterhouse and 1 from a pig raised at the family’s farm. Viral RNA was extracted and reverse transcribed under standard conditions by using the Uni12 (5′-AGCAAAAGCAGG-3′) primer. The viral genomes were amplified by PCR and sequenced by using segment-specific primers (sequences available on request). Genomic sequencing ultimately showed that the 3 isolates were virtually identical, and the sequence of the entire genome of the representative strain A/swine/Jiangsu/40/2011 (Sw/JS/40/11) is available in GenBank (accession nos. JQ319645–JQ319652). Unrooted phylogenetic trees were generated by using MEGA5 software (www.megasoftware.net). The A (H1N1) viruses isolated in this study fell into the European avian-like swine A (H1N1) lineage (Figure 1). The homology of the polymerase basic protein (PB) 2, PB1, polymerase acidic protein, HA, nucleocapsid protein, NA, matrix (M), and nonstructural protein genes between the Sw/JS/40/11 virus and the A/Jiangsu/1/2011 (JS/1/11) virus, which was isolated from the child, were 99.3%, 99.3%, 99.3%, 99.7%, 99.7%, 99.4%, 99.6%, and 99.1%, respectively, indicating that they might have been derived from the same ancestor.

**Figure 2:**
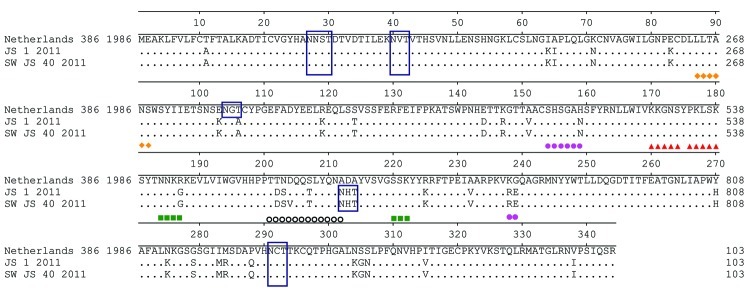
Multiple alignment of hemagglutinin protein sequences. Epitopes Sa, Sb, Ca1, Ca2, and Cb are indicated. Triangle, Sa; circle, Sb; square, Ca1; hexagon, Ca2; diamond, Cb. Putative glycosylation sites are indicated in blue-lined boxes.

The receptor-binding property of the HA protein is a major molecular determinant of host range. The amino acids at sites 190 and 225 of HA are major determinants of the receptor-binding specificity of the A (H1N1) virus, and the mutations E190D and D225E in HA switch the virus receptor-binding specificity from α-2,3–linked sialosides to α-2,6–linked sialosides (6). The Sw/JS/40/11 and JS/1/11 isolates have the amino acids D at site 190 and E at site 225 within the HA protein, which implies that these viruses might preferentially bind to α-2,6–linked sialosides. Potential glycosylation sites (PGSs) also have a major effect on the antigenic and receptor-binding properties of influenza A viruses. Molecular analysis showed that the 2 Jiangsu strains had 5 PGSs in their HA1 proteins, 4 of which were the same as those of the A/Netherlands/386/1986 virus (the cause of the first avian-like SIV infection in a human). Antigenic sites in the H1 HAs, i.e., Sa, Sb, Ca1, Ca2, and Cb, were compared between A/Netherlands/386/1986 and JS/1/11. Amino acid mutations H159N, K238R, and G239E were observed at the Ca2 site; R187G at the Ca1 site; and T202D, N203S, S207T, and A212N at the Sb site. Compared with JS/1/11, the unique mutation D204V, located at the Sb site, which is an antigenic site near the receptor-binding site in influenza virus (7), occurred in the HA1 of Sw/JS/40/11 (Figure 2). No oseltamivir resistance–conferring substitutions (H274Y and N294S) were observed in the NA proteins of the 2 viruses, which suggests that they are sensitive to NA inhibitors (8). The amino acid sequence of the M2 protein of the 2 isolates did not contain the I27T or S31N substitution, characteristic of amantadine resistance in influenza viruses (9,10). The 627K and 701N residues in the PB2 protein contribute to the replication and transmission of avian influenza viruses in mammalian hosts (11–14). Similar to most avian-like A (H1N1) SIVs, both isolates (JS/1/11 and Sw/JS/40/11) had 701N in their PB2 gene.

Chicken antiserum against different subtype H1N1 or H1N2 SIVs were used for antigenic analysis. The Sw/JS/40/11 virus reacted with the antiserum against the classical A (H1N1) SIV (A/swine/Guangdong/6/2010), the triple reassortant A (H1N2) SIV (A/swine/Tianjin/1/2007), influenza A(H1N1)pdm09 (A/swine/Heilongjiang/44/2009), and the avian-like SIV (A/swine/Henan/11/2005) (Table), but not with the antiserum against the human-like A (H1N1) SIV (A/swine/Hebei/15/2009). Antiserum against Sw/JS/40/11 reacted only with the avian-like A (H1N1) SIV and the human-like A (H1N1) SIV, but the HI titers against the human-like A (H1N1) SIV were 4-fold lower than those against the avian-like A (H1N1) SIVs. These results suggest that the H1 subtype SIVs circulating in China differ antigenically.

**Table:** Antigenic analysis of H1 swine influenza viruses, People’s Republic of China*

Virus	HI antibody titers of chicken antiserum against†					
	Classical swine A (H1N1)	Triple-reassortant A (H1N2)	A (H1N1) pdm09	Human seasonal A (H1N1)	Avian-like swine A (H1N1)‡	Avian-like swine A (H1N1)§
Classical swine A (H1N1)	**512**	512	512	¶	8	¶
Triple-reassortant A (H1N2)	1024	**512**	1,024	¶	8	¶
A(H1N1)pdm09	512	512	**1,024**	¶	16	¶
Human seasonal A (H1N1)	16	32	64	**32**	32	64
Avian-like swine A (H1N1)‡	128	64	128	¶	**512**	256
Avian-like swine A (H1N1)§	32	16	64	¶	256	**256**

*Classical swine A (H1N1), A/swine/Guangdong/6/2010; triple-reassortant A (H1N2), A/swine/Tianjin/1/2007; A(H1N1)pdm09, A/swine/Heilongjiang/44/2009; human seasonal A (H1N1), A/swine/Hebei/15/2009. †Antiserum was generated by inoculating specific pathogen-free chickens with an oil-emulsified inactivated vaccine derived from the indicated viruses. Homologous titers are shown in **boldface**. ‡A/swine/Henan/11/2005. §A/swine/Jiangsu/40/2011. ¶HI titer <2.

We investigated antibody responses in 20 serum samples from pigs at the patient’s family farm and the local slaughterhouse. Serologic assays showed that the seroprevalence of antibodies to the avian-like A (H1N1) SIVs was 55% and to classical A (H1N1) SIVs and A(H1N1)pdm09 virus were 25% and 30%, respectively. Furthermore, antibodies against A(H3N2) SIVs were observed but at the low rate of 10%.

## Conclusions

We showed that similar viruses were simultaneously prevalent in a local pig population when a child was infected with an avian-like A (H1N1) SIV. Specifically, isolation of avian-like SIV from a family farm provides direct evidence for the origin of the human infection. No further spread of the Sw/JS/40/2011-like swine strain occurred, according to the limited information available; however, the incident aroused interest in influenza in animals, especially in pigs. Antigenic analysis showed that this avian-like A (H1N1) SIV was antigenically divergent from classical A (H1N1) and human-like A (H1N1) SIVs currently circulating in China, which was further reinforced by the heterogeneity of their genetic relationships. Since early avian-like A (H1N1) SIV isolates in humans, amino acid mutations in the antigenic sites and PGS changes might have altered the antigenic properties in the avian-like A (H1N1) SIV cluster. Our data highlight the need to characterize circulating strains antigenically and genetically through regular influenza virus surveillance.

Pigs can serve as intermediate hosts for influenza viruses to evolve toward efficient replicability in humans. The classical A (H1N1) SIVs and European avian-like A (H1N1) SIVs have circulated worldwide in pigs since 1930 and 1979, respectively, and a classical A (H1N1) SIV emerged in humans as a triple reassortant, causing the 2009 influenza pandemics (15). Although the virulence and transmissibility of the avian-like A (H1N1) SIVs remain to be evaluated, recurrent human infections with avian-like A (H1N1) SIVs suggest that after long-term adaptation in pigs, the avian-like A (H1N1) SIVs already can replicate in humans. After further whole-genome adaptation to the human host or reassortment with other viruses, novel strains bearing the avian-like swine subtype H1N1 HA gene are highly likely to be generated with pandemic potential. Continued surveillance of swine and systemic analysis of swine influenza isolates are clearly needed.

## References

[1] de Jong JC, Claas EC, Osterhaus AD, Webster RG, Lim WL. A pandemic warning? Nature. 1997;389:554. 10.1038/39218 9335492 PMC7095477

[2] de Jong JC, Paccaud MF, de Ronde-Verloop FM, Huffels NH, Verwei C, Weijers TF, Isolation of swine-like influenza A(H1N1) viruses from man in Switzerland and the Netherlands. Ann Inst Pasteur Virol. 1988;139:429–37. 10.1016/S0769-2617(88)80078-9 3214596

[3] Myers KP, Olsen CW, Gray GC. Cases of swine influenza in humans: a review of the literature. Clin Infect Dis. 2007;44:1084–8. 10.1086/512813 17366454 PMC1973337

[4] Adiego Sancho B, Omenaca Teres M, Martinez Cuenca S, Rodrigo Val P, Sanchez Villanueva P, Casas I, Human case of swine influenza A (H1N1), Aragon, Spain, November 2008. Euro Surveill. 2009;14:pii=19120. 19232228

[5] World Organisation for Animal Health. Manual of diagnostic tests and vaccines for terrestrial animals 2010. Chapter 2.8.8. Version adopted by the World Assembly of Delegates of the OIE in May 2010 [cited 2012 Feb 23]. http://www.oie.int/fileadmin/Home/eng/Health_standards/tahm/2.08.08_SWINE_INFLUENZA.pdf

[6] Matrosovich M, Tuzikov A, Bovin N, Gambaryan A, Klimov A, Castrucci MR, Early alterations of the receptor-binding properties of H1, H2, and H3 avian influenza virus hemagglutinins after their introduction into mammals. J Virol. 2000;74:8502–12. 10.1128/JVI.74.18.8502-8512.2000 10954551 PMC116362

[7] Hatta M, Gao P, Halfmann P, Kawaoka Y. Molecular basis for high virulence of Hong Kong H5N1 influenza A viruses. Science. 2001;293:1840–2. 10.1126/science.1062882 11546875

[8] Ives JA, Carr JA, Mendel DB, Tai CY, Lambkin R, Kelly L, The H274Y mutation in the influenza A/H1N1 neuraminidase active site following oseltamivir phosphate treatment leaves virus severely compromised both in vitro and in vivo. Antiviral Res. 2002;55:307–17. 10.1016/S0166-3542(02)00053-0 12103431

[9] Saito R, Sakai T, Sato I, Sano Y, Oshitani H, Sato M, Frequency of amantadine-resistant influenza A viruses during two seasons featuring cocirculation of H1N1 and H3N2. J Clin Microbiol. 2003;41:2164–5. 10.1128/JCM.41.5.2164-2165.2003 12734269 PMC154689

[10] Shiraishi K, Mitamura K, Sakai-Tagawa Y, Goto H, Sugaya N, Kawaoka Y. High frequency of resistant viruses harboring different mutations in amantadine-treated children with influenza. J Infect Dis. 2003;188:57–61. 10.1086/375799 12825171

[11] Subbarao EK, London W, Murphy BR. A single amino acid in the PB2 gene of influenza A virus is a determinant of host range. J Virol. 1993;67:1761–4. 8445709 10.1128/jvi.67.4.1761-1764.1993 PMC240216

[12] Li Z, Chen H, Jiao P, Deng G, Tian G, Li Y, Molecular basis of replication of duck H5N1 influenza viruses in a mammalian mouse model. J Virol. 2005;79:12058–64. 10.1128/JVI.79.18.12058-12064.2005 16140781 PMC1212590

[13] Steel J, Lowen AC, Mubareka S, Palese P. Transmission of influenza virus in a mammalian host is increased by PB2 amino acids 627K or 627E/701N. PLoS Pathog. 2009;5:e1000252. 10.1371/journal.ppat.1000252 19119420 PMC2603332

[14] Gao Y, Zhang Y, Shinya K, Deng G, Jiang Y, Li Z, Identification of amino acids in HA and PB2 critical for the transmission of H5N1 avian influenza viruses in a mammalian host. PLoS Pathog. 2009;5:e1000709. 10.1371/journal.ppat.1000709 20041223 PMC2791199

[15] Gibbs AJ, Armstrong JS, Downie JC. From where did the 2009 ‘swine-origin’ influenza A virus (H1N1) emerge? Virol J. 2009;6:207. 10.1186/1743-422X-6-207 19930669 PMC2787513

